# Supplements of vitamins B9 and B12 affect hepatic and mammary gland gene expression profiles in lactating dairy cows

**DOI:** 10.1186/s12864-016-2872-2

**Published:** 2016-08-15

**Authors:** Bazoumana Ouattara, Nathalie Bissonnette, Melissa Duplessis, Christiane L. Girard

**Affiliations:** 1 Sherbrooke Research and Development Centre, Agriculture and Agri-Food Canada, Sherbrooke, QC J1M 0C8 Canada; 2 Current address: Valacta, Ste-Anne-de-Bellevue, Québec, H9X 3R4 Canada

**Keywords:** Dairy cow, Liver, Mammary gland, Vitamin B9, Vitamin B12, Microarray

## Abstract

**Background:**

A combined supplement of vitamins B9 and B12 was reported to increase milk and milk component yields of dairy cows without effect on feed intake. The present study was undertaken to verify whether this supplementation positively modifies the pathways involved in milk and milk component synthesis. Thus, by studying the transcriptome activity in these tissues, the effect of supplements of both vitamins on the metabolism of both liver and mammary gland, was investigated. For this study, 24 multiparous Holstein dairy cows were assigned to 6 blocks of 4 animals each according to previous 305-day milk production. Within each block, cows were randomly assigned to weekly intramuscular injections of 5 mL of either saline 0.9 % NaCl, 320 mg of vitamin B9, 10 mg of vitamin B12 or a combination of both vitamins (B9 + B12). The experimental period began 3 weeks before the expected calving date and lasted 9 weeks of lactation. Liver and mammary biopsies were performed on lactating dairy cows 64 ± 3 days after calving. Samples from both tissues were analyzed by microarray and qPCR to identify genes differentially expressed in hepatic and mammary tissues.

**Results:**

Microarray analysis identified 47 genes in hepatic tissue and 16 genes in the mammary gland whose expression was modified by the vitamin supplements. Gene ontology (GO) categorizes genes in non-overlapping domains of molecular biology. Panther is one of the online GO resources used for gene function classification. It classifies the 63 genes according to Molecular Function, Biological Process and Protein Class. Most of the biological processes modulated by the vitamin supplements were associated to developmental process, protein metabolic process, transport and response to inflammation. In the liver, most of the genes modulated by the vitamin treatments involved protein metabolic process while developmental process appeared to be more affected by the treatments in mammary gland. Out of 25 genes analysed by qPCR, 7 were validated.

**Conclusion:**

The results indicate that several metabolic processes were modulated by the supplementation of vitamins in early-lactating dairy cows. In addition, the results suggest that the vitamin supplements promoted liver regeneration and reduced catabolism of lipids in early lactation.

**Electronic supplementary material:**

The online version of this article (doi:10.1186/s12864-016-2872-2) contains supplementary material, which is available to authorized users.

## Background

Bacteria present in rumen synthesize B vitamins in generally sufficient amounts to meet their host’s requirements [1]. Notwithstanding, high-producing dairy cows could benefit from vitamin B9 and B12 supplements, especially during the critical period around calving and in early lactation [2–4]. Vitamin B9 plays a major role in DNA synthesis and in *de novo* formation of methyl groups required for the methylation cycle. On the other hand, vitamin B12 is involved in two metabolic pathways: the remethylation cycle and as coenzyme of the methylmalonyl-CoA mutase. The former, closely related to folate metabolism, is required for the regeneration of methionine and tetrahydrofolate, whereas the later allows the entry of propionate in the Krebs cycle and gluconeogenesis [5].

Results from two studies suggest that supplementary vitamin B9 might improve efficiency of nutrient utilization, especially for milk protein synthesis [6, 7]. This observation is supported by the observation that in vitro hormonal stimulation of milk protein synthesis by mammary gland explants of dairy cows increased expression of 28 genes; among them, 2 genes related to folate metabolism, *FOLR1* and *ALDH1L1* [8]. The former is a folate transporter allowing the entry of 5-methyl-tetrahydrofolate into the cells whereas the latter is involved in purine synthesis. As vitamin B9 supplement increases milk protein synthesis, it would be interesting to investigate whether similar transcriptomic process takes place in vivo when vitamin supplements are provided to lactating cows.

A combined supplement of vitamins B9 and B12 given during the *peripartum* period and in early lactation altered energy partitioning during the first weeks of lactation as compared to control cows [3, 4, 9]. However, the mode of action of this supplement is not fully elucidated. We observed that a combined supplement of vitamins B9 and B12 increases whole-body rate of appearance of glucose which is the sum of glucose from portal absorption, glycogenolysis, and gluconeogenesis [3]. Propionate originating from rumen fermentation is the major precursor of glucose in cows [10, 11] and contributes up to 60 % of glucose flux rate [12, 13]. For ruminants, the glucose, essential for synthesis of milk lactose, is mostly provided by liver (up to 90 %) through gluconeogenesis [14].

It has been shown that at the onset of lactation, the liver as well as the mammary gland undergo numerous adaptations to support milk synthesis [15]. In early lactation, feed intake is not sufficient to meet the nutrient demand for milk production, leading to a negative energy balance [15]. Furthermore, the liver undergoes extensive physiological and biochemical changes mediated by significant alterations in hepatic gene expression in an attempt to re-establish metabolic homeostasis and to counteract the adverse effects of negative energy balance [16]. In addition, during this period, the mammary gland is actively remodeling. In fact, by investigating the transcriptional response of the mammary gland during early lactation, Connor et al. [17] observed changes in mammary expression of genes involved in cell proliferation, cellular remodeling, and nutrient transport.

Therefore, the aim of the present project was to profile the genes for which expression undergoes major changes in hepatic and mammary tissues of lactating dairy cows according to vitamins B9 and B12 supply.

## Results

At week 9 of lactation, there was no treatment effect (*P* ≥ 0.1) on dry matter intake, milk production, milk total solid yields as well as milk component contents. Dry matter intake, milk production, milk total solid yields, milk fat, protein and lactose contents averaged 18.8 (SE 1.9) kg/d, 34.7 (SE 2.9) kg/d, 3.92 (SE 0.33) kg/d, 36.0 (SE 2.0) g/kg, 30.2 (SE 1.4) g/kg, and 46.9 (SE 6.0) g/kg, respectively. During the seven weeks following calving, body condition score losses tended to be reduced (interaction vitamin × time, *P* = 0.10) in cows receiving the vitamin B9 supplements, alone or combined with vitamin B12. Plasma concentrations of non-esterified fatty acids and β-hydroxybutyrate were also lower (*P* ≤ 0.06), averaging 175 and 243 μM (SEM 17) and 0.70 and 0.77 mM (SEM 0.03) for cows receiving or not receiving supplementary vitamin B9, respectively.

**Fig. 1:**
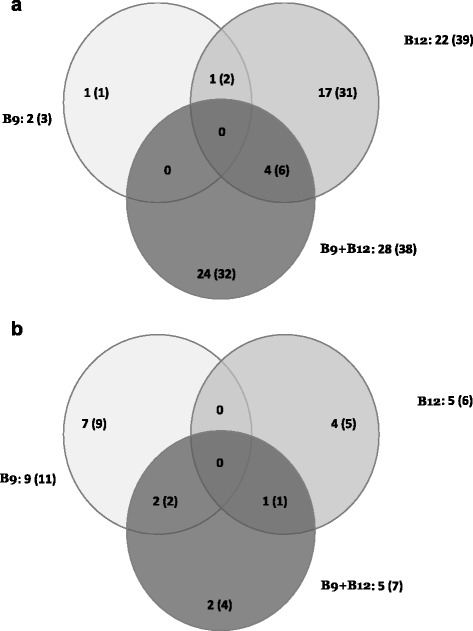
Number of genes (and probes) influenced by the vitamin supplementation. Supplements of vitamin B9 (B9), vitamin B12 (B12) or both vitamins (B9 + B12) were given to dairy cows. The Venn diagram shows the number of significant genes. The number of probes is bracketed “()”. (FDR ≤ 0.05) with more than 2-fold change between the control group and each treatment group in (**a**) hepatic tissue and (**b**) mammary tissue

**Fig. 2:**
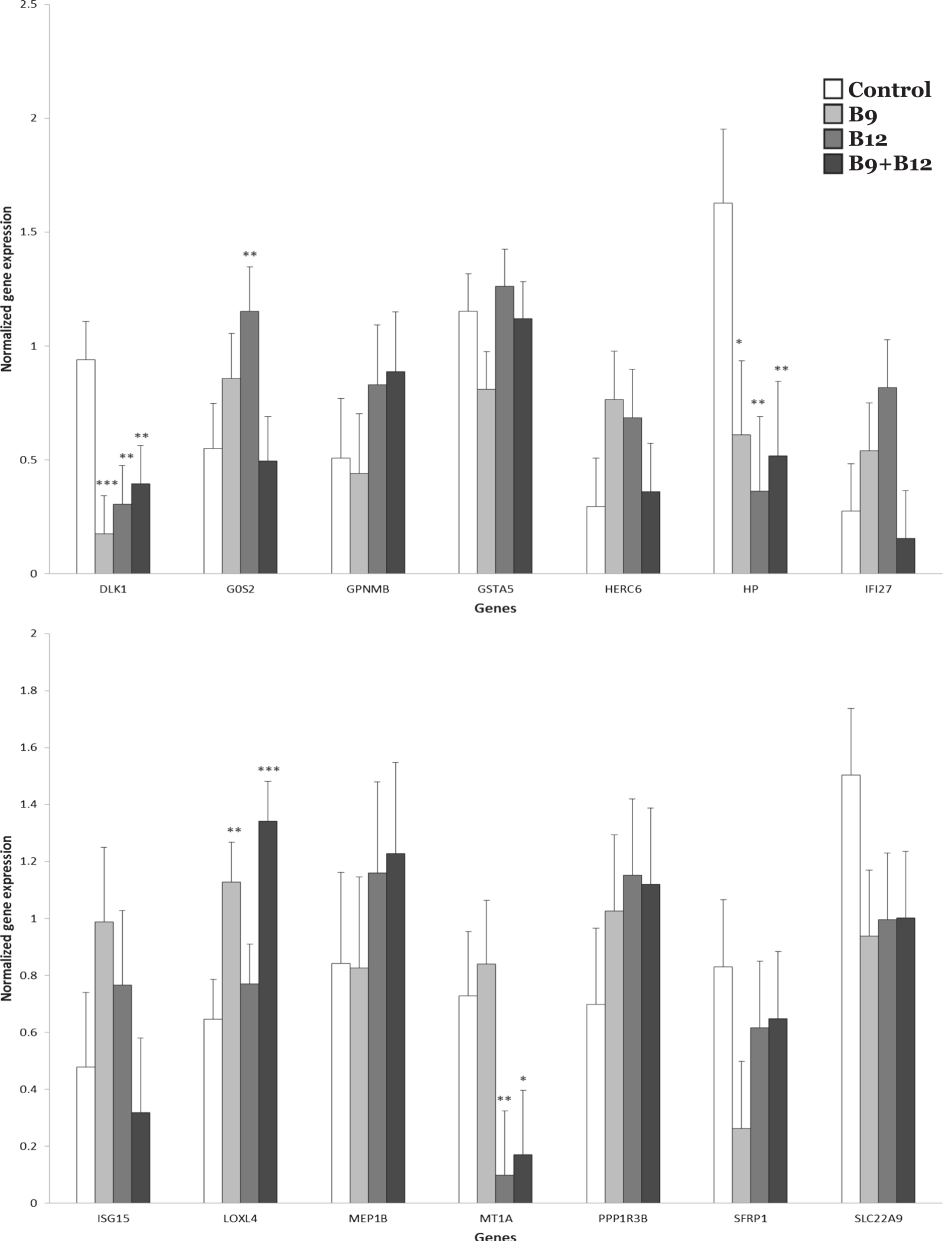
Expression of genes measured by quantitative PCR in the liver of the dairy cows that received either no vitamin supplement: Control: no vitamin supplement; vitamin B9 supplement (B9); vitamin B12 supplement (B12) or a combined supplement of vitamins B9 and B12 (B9 + B12). Means different from the control treatment are indicated by *** when *P* value ≤ 0.01, ** when *P* values were between 0.01 and 0.05 and * when there was a trend with *P* values between 0.05 and 0.1

The microarray analysis revealed that, as compared to control cows receiving no vitamin supplement, the vitamin treatments significantly changed (FDR ≤ 0.05) the expression of some genes by more than 2-fold in hepatic and mammary tissues (Fig. 1 a and b, respectively). The expression of 47 genes in hepatic tissue was modified by at least one of the vitamin treatment compared to control cows (Fig. 1a). From this panel of 47 genes, 41 could be assigned to a biological process using the Panther classification system (Tables 1 and 2). Out of these 47 genes, expression of 14 genes was analyzed by qPCR (Fig. 2). Although nine false positive genes were observed, five genes were confirmed to be differentially expressed in the group of cows supplemented with vitamins.

**Table 1:** Gene Ontology annotations of the differentially expressed hepatic genes with the Panther Classification System^a^

Treatment	Gene symbol	Gene name	Treatment effect	Molecular function	Biological process	Protein class
B9						
*DLK1*		Delta-like 1 homolog (Drosophila)	Down-regulated	receptor activity extracellular matrix structural constituent receptor binding	transcription from RNA polymerase II promoter cell communication ectoderm development nervous system development intracellular protein transport receptor-mediated endocytosis regulation of transcription from RNA polymerase II promoter	membrane-bound signaling molecule receptor extracellular matrix structural protein
*MYOM1*		Myomesin-1	Down-regulated	protein kinase activity structural constituent of cytoskeleton protein binding small GTPase regulator activity guanyl-nucleotide exchange factor activity	protein phosphorylation cell communication cell adhesion muscle contraction mesoderm development muscle organ development regulation of catalytic activity	non-receptor serine/threonine protein kinase non-receptor serine/threonine protein kinase guanyl-nucleotide exchange factor actin family cytoskeletal protein cell adhesion molecule
B12						
*HERC6*		HECT and RLD domain containing E3 ubiquitin protein ligase family member 6	Up-regulated	ligase activity	catabolic process cellular protein modification process proteolysis cellular process	ubiquitin-protein ligase
*SESN2*		Sestrin-2	Up-regulated	oxidoreductase activity peroxidase activity	metabolic process cell cycle	Peroxidase
*GPNMB*		glycoprotein (transmembrane) nmb	Up-regulated	receptor binding	cellular process	membrane-bound signaling molecule cell adhesion molecule
*IFI6*		Interferon alpha-inducible protein 6	Up-regulated	Unassigned	Unassigned	Unassigned
*PPP1R3B*		Protein phosphatase 1 regulatory subunit 3B	Up-regulated	phosphatase activity protein binding phosphatase regulator activity	glycogen metabolic process regulation of catalytic activity	phosphatase modulator
*ID1*		ID1 protein	Up-regulated	sequence-specific DNA binding transcription factor activity	transcription from RNA polymerase II promoter regulation of transcription from RNA polymerase II promoter	transcription factor
*IFI27*		interferon, alpha-inducible protein 27	Up-regulated	Unassigned	Unassigned	Unassigned
*MEP1B*		meprin A, beta	Up-regulated	oxidoreductase activity serine-type peptidase activity metallopeptidase activity receptor activity lipid transporter activity transmembrane transporter activity receptor binding enzyme regulator activity	immune system process proteolysis synaptic transmission cell-cell adhesion visual perception sensory perception ectoderm development mesoderm development skeletal system development angiogenesis nervous system development heart development blood coagulation lipid transport intracellular protein transport endocytosis vitamin transport regulation of catalytic activity	transporter apolipoprotein membrane-bound signaling molecule receptor metalloprotease serine protease oxidase metalloprotease serine protease extracellular matrix protein enzyme modulator cell adhesion molecule
*FUT5*		fucosyltransferase 5 (alpha (1,3) fucosyltransferase	Up-regulated	transferase activity, transferring glycosyl groups	protein glycosylation	glycosyltransferase
*G0S2*		G0/G1switch 2	Up-regulated	Unassigned	Unassigned	Unassigned
*CDK5R1*		Cyclin-dependent kinase 5 activator 1	Up-regulated	kinase activity protein binding kinase activator activity kinase regulator activity	protein phosphorylation cell cycle regulation of catalytic activity	kinase activator
*ISG15*		Ubiquitin-like protein ISG15	Up-regulated	structural constituent of ribosome nucleic acid binding	proteolysis	ribosomal protein
LOC515676		NFX1-type zinc finger-containing protein 1-like	Up-regulated	peptidase activity protein binding serine-type endopeptidase inhibitor activity	proteolysis regulation of catalytic activity	serine protease inhibitor
*SPP1*		Osteopontin	Up-regulated	cytokine activity	immune system process cellular process cell adhesion cellular component morphogenesis cellular component organization	cytokine extracellular matrix protein defense/immunity protein cell adhesion molecule
*DLK1*		Delta-like 1 homolog (Drosophila)	Down-regulated	receptor activity extracellular matrix structural constituent receptor binding	transcription from RNA polymerase II promoter cell communication ectoderm development nervous system development intracellular protein transport receptor-mediated endocytosis regulation of transcription from RNA polymerase II promoter	membrane-bound signaling molecule receptor extracellular matrix structural protein
*MYOM1*		Myomesin-1	Down-regulated	protein kinase activity structural constituent of cytoskeleton protein binding small GTPase regulator activity guanyl-nucleotide exchange factor activity	protein phosphorylation cell communication cell adhesion muscle contraction mesoderm development muscle organ development regulation of catalytic activity	non-receptor serine/threonine protein kinase non-receptor serine/threonine protein kinase guanyl-nucleotide exchange factor actin family cytoskeletal protein cell adhesion molecule
*MT1E*		Metallothionein MT1E	Down-regulated	Unassigned	Unassigned	Unassigned
*NEFH*		ortholog Uncharacterized protein (Fragment) NEFH ortholog	Down-regulated	Unassigned	Unassigned	Unassigned
*SFRP1*		Secreted frizzled-related protein 1	Down-regulated	receptor activity protein binding	reproduction cell communication single-multicellular organism process nervous system development response to stimulus regulation of biological process	signaling molecule G-protein coupled receptor
*KIAA1324*		KIAA1324 ortholog	Down-regulated	Unassigned	Unassigned	Unassigned
*MGC126945*		Uncharacterized protein	Down-regulated	receptor activity	B cell mediated immunity antigen processing and presentation cellular defense response	immunoglobulin receptor superfamily immunoglobulin receptor superfamily major histocompatibility complex antigen
*MT1A*		Metallothionein-1A MT1A	Down-regulated	Unassigned	Unassigned	Unassigned
B9 + B12						
*SAA3*		Serum amyloid A protein	Down-regulated	lipid transporter activity transmembrane transporter activity	immune system process lipid transport	transporter apolipoprotein defense/immunity protein
LOC100126815		MHC class I-like family A1	Down-regulated	receptor activity	B cell mediated immunity antigen processing and presentation cellular defense response	immunoglobulin receptor superfamily immunoglobulin receptor superfamily major histocompatibility complex antigen
*ACMSD*		2-amino-3-carboxymuconate-6-semialdehyde decarboxylase	Down-regulated	Unassigned	Unassigned	Unassigned
*THRSP*		THRSP protein	Down-regulated	Unassigned	Unassigned	Unassigned
*MGC126945*		Uncharacterized protein	Down-regulated	receptor activity	B cell mediated immunity antigen processing and presentation cellular defense response	immunoglobulin receptor superfamily immunoglobulin receptor superfamily major histocompatibility complex antigen
*MT1A*		Metallothionein-1A MT1A	Down-regulated	Unassigned	Unassigned	Unassigned
*C4H7orf57*		chromosome 4 open reading frame, human C7orf57	Down-regulated	Unassigned	Unassigned	Unassigned
*CACNA2D1*		calcium channel, voltage-dependent, alpha 2/delta subunit	Down-regulated	cation transmembrane transporter activity	cation transport protein targeting	Unassigned
*GSTA5*		Glutathione S-transferase	Down-regulated	Unassigned	Unassigned	Unassigned
LOC509034		feline leukemia virus subgroup C receptor-related protein 2-like	Down-regulated	transmembrane transporter activity	transport	transporter
*NHEDC1*		solute carrier family 9, subfamily B (cation proton antiporter 2), member 1	Down-regulated	Unassigned	Unassigned	Unassigned
*SH3YL1*		SH3 domain-containing YSC84-like protein 1	Down-regulated	structural constituent of cytoskeleton actin binding	cellular process	non-motor actin binding protein
*HP*		Haptoglobin	Down-regulated	serine-type peptidase activity calcium ion binding calmodulin binding calcium-dependent phospholipid binding	gamete generation complement activation proteolysis cellular process blood circulation response to stress blood coagulation	serine protease serine protease complement component annexin calmodulin
*NGEF*		neuronal guanine nucleotide exchange factor	Down-regulated	Unassigned	Unassigned	Unassigned
*LGALS3*		Lectin, galactoside-binding, soluble, 3	Up-regulated	receptor binding	cellular process	signaling molecule cell adhesion molecule
LOC524810		IgM	Up-regulated	Unassigned	Unassigned	Unassigned
*LOXL4*		Lysyl oxidase homolog 4	Up-regulated	oxidoreductase activity serine-type peptidase activity receptor activity	macrophage activation apoptotic process proteolysis cell communication cell-cell adhesion neurological system process cellular defense response extracellular transport negative regulation of apoptotic process	receptor serine protease oxidase serine protease
*PYCR1*		Pyrroline-5-carboxylate reductase 1, mitochondrial	Up-regulated	oxidoreductase activity	cellular amino acid biosynthetic process	reductase
*MSMB*		Uncharacterized protein	Up-regulated	hormone activity		peptide hormone
*IGLL1*		immunoglobulin lambda-like polypeptide 1	Up-regulated	antigen binding	B cell mediated immunity hemopoiesis response to stimulus	Immunoglobulin
*MT1E*		Metallothionein MT1E	Down-regulated	Unassigned	Unassigned	Unassigned
*GPNMB*		glycoprotein (transmembrane) nmb	Up-regulated	receptor binding	cellular process	membrane-bound signaling molecule cell adhesion molecule

All genes differentially expressed in liver were classified using the Gene Ontology annotations with the Panther Classification System. Some of these genes were not found by Panther genes list analysing system and some could not be classified. Only three ontology categories are presented: Molecular Function, Biological Process and Protein Class

^a^Panther gene list classification system of the genes differentially expressed in hepatic tissue of cows receiving vitamin supplements (vitamin B9 alone: B9, vitamin B12 alone: B12 or both vitamins: B9 + B12) as compared to no vitamins treatment (Control)

**Table 2:** Identification of the major biological processes of the genes differentially expressed in hepatic tissue^a^

Biological process		Genes	
Level 1	Level 2/3	Symbol	Fold Change
apoptotic process	negative regulation of apoptotic process	*LOXL4*	1.6
biological adhesion	cell adhesion	*MYOM1* *SPP1* *MEP1B* *LOXL4*	−2.5 0.9 1.3 1.6
biological regulation	regulation of biological process	*DLK1* *SFRP1* *ID1* *LOXL4*	−2.5 −1.8 1.1 1.6
	regulation of molecular function	*MYOM1* *CDK5R1* *PPP1R3B* *MEP1B* LOC515676	−2.5 0.8 1.1 1.3 1.9
cellular component organization or biogenesis	cellular component organization	*SPP1*	0.9
cellular process	cell communication	*DLK1* *MYOM1* *SFRP1* *MEP1B* *LOXL4*	−2.5 −2.5 −1.8 1.3 1.6
	cell cycle	*CDK5R1* *SESN2*	0.8 0.9
developmental process	anatomical structure morphogenesis	*SPP1*	0.9
	death	*LOXL4*	1.6
	ectoderm development	*DLK1* *MEP1B*	−2.5 1.3
	mesoderm development	*MYOM1* *MEP1B*	−2.5 1.3
	system development	*DLK1* *MYOM1* *SFRP1* *MEP1B* *IGLL1*	−2.5 −2.5 −1.8 1.3 2.1
immune system process	antigen processing and presentation	LOC100126815 *MGC126945*	−2.4 −1.8
	immune response	LOC100126815 *MGC126945* *HP* *IGLL1*	−2.4 −1.8 −1.6 2.1
	macrophage activation	*LOXL4*	1.6
localization	transport	*DLK1* LOC509034 *SAA3* *CACNA2D1* *MEP1B* *LOXL4*	−2.5 −1.5 −1.1 −1.0 1.3 1.6
metabolic process	catabolic process	*HERC6*	1.8
	carbohydrate metabolic process	*PPP1R3B*	1.1
	cellular amino acid metabolic process	*PYCR1*	1.5
	nucleobase-containing compound metabolic process	*DLK1* *ID1*	−2.5 1.1
	protein metabolic process	*MYOM1* *HP* *CDK5R1* *MEP1B* *LOXL4* *FUT5* *HERC6* LOC515676 *ISG15*	−2.5 −1.6 0.8 1.3 1.6 1.6 1.8 1.9 2.5
multicellular organismal process	single-multicellular organism process	*MYOM1* *SFRP1* *HP* *MEP1B* *LOXL4*	−2.5 −1.8 −1.6 1.3 1.6
reproduction	gamete generation	*HP*	−1.6
response to stimulus	cellular defense response	LOC100126815 *MGC126945* *LOXL4*	−2.4 −1.8 1.6
	immune response	LOC100126815 *MGC126945* *HP* *IGLL1*	−2.4 −1.8 −1.6 2.1
	response to external stimulus	*HP* *MEP1B*	−1.6 1.3
	response to stress	*HP*	−1.6
Not found		*C20H5orf49* LOC789904 *SLC22A9* *CFH* *SLC26A10* *SAA2* *M*-*SAA3* LOC100847494 LOC100335975 LOC617654	−1.9 −1.8 −1.7 −1.5 −1.4 −1.2 −1.1 −0.9 1.4 4.2
Unassigned		*C4H7orf57* *MT1E* *MT1A* *THRSP* *KIAA1324* *NGEF* *GSTA5* *ACMSD* *NHEDC1* *NEFH* *G0S2* LOC524810 *IFI27* *IFI6*	−4.4 −3.4 −3.3 −1.7 −1.6 −1.4 −1.3 −1.2 −1.1 −1.1 1.9 2.6 3.3 3.7

All genes modulated in the liver by the vitamins treatment, including those that were not found or classified by the Panther system are listed. The fold change observed by microarray analysis varies from −4.4 to 4.2 and only significantly (FDR ≤ 0.05) expressed genes (compared to control) are shown

^a^Identification of the major biological processes of the genes differentially expressed in hepatic tissue of cows receiving vitamin supplements (vitamin B9 alone: B9, vitamin B12 alone: B12 or both vitamins: B9 + B12) as compared to no vitamins treatment (Control)

The expression of two genes was significantly modified in liver by the B9 treatment as illustrated in the Venn diagram (Fig. 1a). One gene that also significantly down-regulated by the B12 treatment was confirmed by qPCR for these cows. This repression of *DLK1* in liver was down-regulated by the three vitamin treatments (Fig. 2). The B12 treatment had the greatest impact on the liver with 22 genes (totalizing 39 significant probes; Fig. 1a) whose expression level differed by more than 2-fold compared to control (Table 2).

Among the 22 genes affected by the B12 treatment, four genes were also influenced in cows receiving the B9 + B12 treatment: the methallothioneins 1A (*MT1A*) and 1E (*MTIE*), the transmembrane glycoprotein (*GPNMB*), and an uncharacterized protein (*MGC126945*) (Table 1). Expression of two of these genes (*MT1A* and *GPNMB*) was studied by qPCR (Fig. 2). This analysis confirmed the repression of *MTIA* in liver of cows receiving B12 and B9 + B12 treatments. Results from the microarray analysis indicated an up-regulation of the expression of *GPNMB* with both B12 and B9 + B12 treatments. The qPCR analysis showed a numerical but not statistically significant increase with these treatments probably due to the large variation in the expression for this gene among the limited number of animals of this study (Fig. 2). Among the other 17 genes affected by the B12 treatment, the expression of seven genes were studied by qPCR but no effect were confirmed for 6 of them (*HERC6*, *IF127*, *ISG15*, *MEP1B*, *PPP1R3B* and *SFRP1*). Only the expression of the G0/G1switch 2 gene (*G0S2*) was up-regulated in liver of cows receiving the B12 treatment as compared to control (Fig. 2).

In addition to these four genes influenced by B12 treatment, alone and in combination with B9, 24 other genes were affected when both vitamins were administrated simultaneously to the cows; four genes were studied by qPCR. Two of them, namely the haptoprotein (*HP*) and lysyl oxidase-like 4 (*LOXL4*), were confirmed as being respectively down and up-regulated by the combined treatment. Nevertheless, the expression of *LOXL4* was also up-regulated in liver of B9 cows whereas, the expression of *HP* was down-regulated by all vitamin treatments (Fig. 2). No difference in expression of *GSTA5* and *SLC22A9* could be detected.

**Fig. 3:**
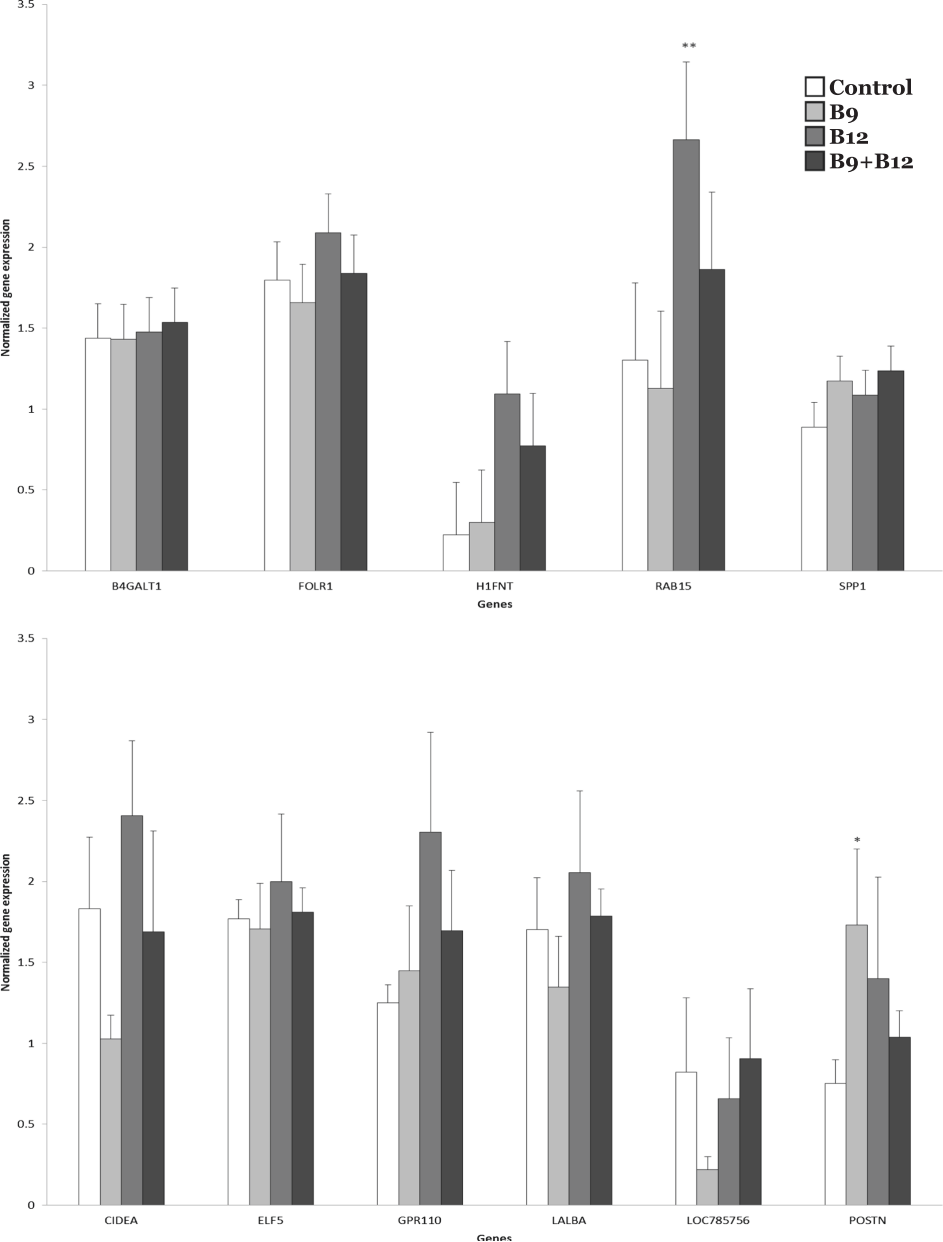
Expression of genes in mammary gland measured by quantitative PCR according to treatments. Control: no vitamin supplement; B9: vitamin B9 supplement; B12: vitamin B12 supplement; B9 + B12: combined supplement of vitamins B9 and B12. Means different from the control treatment are indicated by *** when *P* value ≤ 0.01, ** when *P* values were between 0.01 and 0.05 and * when there was a trend with *P* values between 0.05 and 0.1

In mammary gland, the B9 treatment modified the expression of nine genes by 2-fold as compared to cows receiving no vitamin supplement. The B12 treatment affected five genes and the expression of five genes was influenced by the B9 + B12 treatment (Fig. 1b). The B9 + B12 shared one gene with the B12 treatment. Based on results from the microarray analysis, three genes whose expression was modified by the B9 treatment (Fig. 1b), cell death-inducing DFFA-like effector a (*CIDEA*), the androgen binding protein beta-like (LOC785756), and the periostin (*POSTN*) genes were studied by qPCR in addition to some candidate genes (Fig. 3). Out of the 11 genes analyzed by qPCR, two genes were confirmed; then, the percentage of false positive in the mammary was 82 %. *RAB15* and *POSTN* were significantly up-regulated respectively by the B12 and B9 as compared to control (Fig. 3). Out of the 16 genes affected in the mammary gland by vitamin supplements, 13 were classified using Panther classification system (Table 3).

**Table 3:** Panther gene list classification system for the genes differentially expressed in mammary gland tissue^a^

Treatment	Gene symbol	Gene name	Treatment regulation effect	Molecular Function	Biological Process	Protein Class
B9						
*POSTN*		Periostin	Up-	receptor binding	cell communication cell-matrix adhesion visual perception sensory perception mesoderm development skeletal system development muscle organ development	signaling molecule cell adhesion molecule
*FUT5*		Alpha-(1,3)-Fucosyltransferase	Up-	transferase activity, transferring glycosyl groups	protein glycosylation	glycosyltransferase
*ATP6V1G3*		V-Type Proton Atpase Subunit G 3	Up-	hydrolase activity cation transmembrane transporter activity proton-transporting ATP synthase activity, rotational mechanism	nucleobase-containing compound metabolic process cation transport	ATP synthase hydrolase
LOC785756		androgen binding protein beta-like	Down-	Unassigned	Unassigned	Unassigned
*MGC126945*		uncharacterized protein MGC126945	Down-	receptor activity	B cell mediated immunity antigen processing and presentation cellular defense response	immunoglobulin receptor superfamily major histocompatibility complex antigen
*CIDEA*		cell death-inducing DFFA-like effector a	Down-	Unassigned	induction of apoptosis	Unassigned
B12						
*GPR110*		G-Protein Coupled Receptor 110-Related	Up-	G-protein coupled receptor activity	spermatogenesis immune response synaptic transmission neurotransmitter secretion mesoderm development heart development response to stress intracellular protein transport synaptic vesicle exocytosis	G-protein coupled receptor antibacterial response protein
*IRX6*		iroquois homeobox 6	Up-	sequence-specific DNA binding transcription factor activity sequence-specific DNA binding transcription factor activity	transcription from RNA polymerase II promoter ectoderm development nervous system development regulation of transcription from RNA polymerase II promoter	homeobox transcription factor nucleic acid binding
*RAB15*		Ras-related protein Rab-15	Up-	Unassigned	Unassigned	Unassigned
B9 + B12						
LOC509034		feline leukemia virus subgroup C receptor-related protein 2-like	Down-	transmembrane	transporter activity transport	Transporter
*IDO1*		indoleamine 2,3-dioxygenase 1	Down-	Unassigned	Unassigned	Unassigned
*SECTM1*		SECTM1 protein	Down-	Unassigned	Unassigned	Unassigned

Differentially expressed genes in mammary tissue were assigned to gene ontology pathways using the Panther tool. Some genes were not found in Panther genes list. Furthermore, among the genes ID found by the PANTHER system, some were not classified to a category. Only 3 genes ontology categories are presented: Molecular Function, Biological Process and Protein Class

^a^Classification with the Panther gene list classification system of the genes differentially expressed in mammary tissue following supplementation with vitamin B9 (B9), vitamin B12 (B12) or both (B9 + B12) as compared to no vitamin treatment (Control)

As shown in Tables 1 and 3, some of the genes identified by microarray in liver and mammary gland were annotated and clustered into three major gene ontology groups: Protein Class, Molecular Function and Biological Process. In Tables 2 and 4, gene expression clusters are categorized within biological processes (levels 1, 2 and 3). This allows looking for statistically over- and under-represented biological process categories among the genes. Most of the genes modulated in the liver by at least one of the vitamin supplements were associated to developmental process, protein metabolic process, transport and immune response (Table 2). However, protein metabolic process was over-represented (with 9 genes: *MYOM1*, *HP*, *CDK5R1*, *MEP1B*, *LOXL4*, *FUT5*, *HERC6*, LOC515676 and *ISG15*) in the gene list affected by treatments in the liver (Table 2). In the mammary gland, where very few genes were affected by the vitamin treatments, the over-represented biological process was developmental process involving 4 genes: *CIDEA*, *POSTN*, *GPR110* and *IRX6* (Table 4).

**Table 4:** Identification of the major biological processes of the genes differentially expressed in mammary gland tissue^a^

Biological process		Genes	
Level 1	Level 2/3	Symbol	Fold change
apoptotic process	induction of apoptosis	*CIDEA*	−1.9
biological adhesion	cell adhesion	*POSTN*	1.1
biological regulation	regulation of biological process	*IRX6*	1.7
cellular process	cell communication	*POSTN* *GPR110*	1.1 1.3
developmental process	death	*CIDEA*	−1.9
	ectoderm development	*IRX6*	1.7
	mesoderm development	*POSTN* *GPR110*	1.1 1.3
	system development	*POSTN* *GPR110* *IRX6*	1.1 1.3 1.7
immune system process	antigen processing and presentation	*MGC126945*	−1.5
	immune response	*MGC126945* *GPR110*	−1.9 1.3
localization	transport	*ATP6V1G3* LOC509034 *GPR110*	−0.9 −0.6 1.3
metabolic process	nucleobase-containing compound metabolic process	*ATP6V1G3* *IRX6*	−0.9 1.7
	protein metabolic process	*FUT5*	3.6
multicellular organismal process	single-multicellular organism process	*POSTN* *GPR110*	1.1 1.3
reproduction	gamete generation	*GPR110*	1.3
response to stimulus	cellular defense response	*MGC126945*	−1.9
	immune response	*MGC126945* *GPR110*	−1.9 1.3
	response to stress	*GPR110*	1.3
Not found		LOC751574 *VAV1* LOC614268 *SAA2* *C10H14orf53* *H1FNT*	−1.3 −1.2 −0.9 −0.9 1.4 4.9
Unassigned		LOC785756 *IDO1* *SECTM1* *RAB15*	−2.1 −1.2 −0.9 2.0

All genes modulated in the mammary tissue by the vitamins treatment, including those that were not found or classified by the Panther system are listed. The Fold Change, as using FlexArray microarray analysis, varies from −1.9 to 4.9 and only significantly (FDR ≤ 0.05) expressed genes (compared to control) are shown

^a^Identification of the major biological processes of the genes differentially expressed in mammary tissue following supplementation with vitamin B9 (B9), vitamin B12 (B12) or both vitamins (B9 + B12) as compared to no vitamin treatment (Control)

Gene symbols were uploaded to the Panther workspace in order to classify the genes of interest by selecting the *Bos taurus* reference gene list based on the selected organism [18]. Notwithstanding, some genes in both tissues (liver and mammary gland) could not be assigned to any biological process or molecular function category by Panther classification system (Tables 1, 2, 3 and 4). It is also possible that there is no experimental data to support their biological annotation.

## Discussion

Microarray analysis was used to investigate the effects of vitamin B9 and B12 supplements given alone or in combination during the *peripartum* early lactation period on liver and mammary gland tissues. The expression of only a limited number of genes was modulated by the vitamin treatments in both tissues, which suggests a subtle vitamin effect on the tissue metabolism that would have been better characterized using a larger population. Notwithstanding, the qPCR analyses show that, in liver, expression of keys genes, such as *DLK1*, *LOXL4*, *G0S2*, *GSTA5*, *HP*, *MT1A*, *IFI27* and *SFRP1* was modulated at different extents (significant effects for five genes and a strong trend for three genes) by the vitamin treatments. Delta-like 1 homolog (DLK1/Pref-1) is a surface marker of hematopoietic progenitor cells (HPCs) associated with less differentiated hepatocellular phenotypes [19] and it has been shown to act in vitro as an inhibitor of Notch signaling [20, 21] to promote liver regeneration [21]. Interestingly, *DLK1* is an imprinted gene which is involved in lipid metabolic reprogramming [22]. An increased concentration of this biomarker in blood serum is associated with hepatic cancer [23] whereas downregulation of *DLK1* expression through an epigenetic mechanism contributes to attenuate liver disease [24]. Because vitamin B9 plays a major role in *de novo* formation of methyl groups and vitamin B12 is required for the remethylation cycle, we can speculate that *DLK1* expression could also be repressed in the liver of the lactating cows through an epigenetic mechanism. Because *DLK1* suppresses glucose production and fatty acid synthesis and oxidation in hepatocytes [25], supplementation of both vitamins B9 and B12 might increase liver metabolism through a genomic imprinting mechanism which negatively impacts the *DLK1* pathway.

Lysyl oxidase-like member 4 (*LOXL4*), a matrix-remodeling enzymes, is extracellularly secreted and significantly contributes to ECM deposition [26]. Activity of lysyl oxidase (LOX) and LOX like proteins are correlated to collagen and elastin deposition and, in adult mammals, are essential to tissue maintenance [27]. Recent studies have provided compelling evidence that *G0S2* is abundantly expressed in metabolically active tissues such as liver, and acts as a molecular brake on triglyceride catabolism [28]. Triglyceride hydrolase activity of adipose triglyceride lipase can be selectively inhibited by *G0S2* [28]. Hence, increasing the expression of *G0S2* decreased lipolysis [29] which is supported by the reduction of plasma concentrations of non-esterified fatty acids in cows receiving vitamin B9 supplements, alone or combined with vitamin B12 in the present study. Although increased *IFI27* expression was not significant, the pattern was highly similar to *G0S2* thus suggesting a similar B9 supplement effect on the liver for this gene. Expression of the alpha-inducible protein 27 (*IFI27*) is up-regulated during inflammatory wound repair process [30] and expression of this gene also alters immune response and mitochondrial function [31]. Interestingly, *DLK1* locus expression is also associated with a restriction of the mitochondrial metabolism [32]. These gene expression patterns support the hypothesis that both vitamins B9 and B12 improve the hepatic function which might reduce metabolic stress during the transition period and early lactation of dairy cows. This is further supported by the marked reduction of the *HP* and *MT1A* genes. The liver is the major site for the synthesis of acute phase proteins including haptoglobin (*HP*) and metallothionein 1A (*MT1A*) [30]. During stress response, it is reported that physiological processes aimed on redistribution of energy utilization in specific organs stimulating mobilization of body reserves. In mammary gland, administration of the three vitamin treatments had a very limited effect on gene expression as described above. Interestingly, in the present study, whereas vitamin treatments had no effect on milk total solids yield and dry matter intake, vitamin B9 supplements, given alone or in combination with vitamin B12, decreased body condition score losses during the first weeks of lactation as well as plasma concentrations of non-esterified fatty acids and β-hydroxybutyrate [9] suggesting an improvement in energy balance for these cows.

In the present study, all the genes that have their expression affected by the vitamin treatments in the liver, are involved in tissue repair, resorption of inflammation and lipid metabolism although no mode of action can be clearly identified. During the first weeks of lactation, dairy cows are generally in negative energy balance because nutrient intake increases less rapidly than nutrient demand for initiation of lactation which leads to mobilization of body reserves. Cows are losing body condition score and non-esterified fatty acids are released from adipose tissues and their plasma concentrations increased. During this period, dairy cows are also prone to liver steatosis because hepatic uptake of non-esterified fatty acids is greater than the amounts oxidized or secreted by the liver [33]. Accumulation of lipids in liver affects integrity and function of hepatic cells [33]; in response, liver parenchymal cells produce an acute-phase glycoprotein haptoglobulin [34]. Because a decrease in *DLK1* can improved fatty acid oxidation from hepatocytes [25], an improved β-oxidation of non-esterified fatty acids in liver could help to reduce ketone body formation and plasma concentrations of β-hydroxybutyrate. The improvement in energy balance observed in cows receiving supplementary vitamin B9, alone or combined with vitamin B12, likely reduced the liver burden caused by mobilization of body fat reserves which could explain the changes in hepatic gene expression described above. For instance, the increase of *LOXL4* and *G0S2* strongly support that these treatments protect body fat from catabolism. Prevention of liver damage or improved liver performance is not only important for maintaining liver function but also for general health of high-yielding dairy cows.

## Conclusion

In the present study, a supplement of vitamin B9, given alone or in combination with vitamin B12, reduced mobilization of body fat reserves and hepatic lipid catabolism in early lactation. Changes in expression of genes described above support the hypothesis that hepatic tissue integrity in early lactation was improved by these vitamin supplements.

## Methods

### Animals and treatments

For the purpose of the present study, biopsies of hepatic and mammary tissues were taken from 24 multiparous Holstein cows from the dairy herd at the Agriculture and Agri-Food Canada Research Centre (Sherbrooke, Quebec, Canada) at the end of a larger study [9]. Care of cows followed the guidelines of the National Farm Animal Care Council (2009) [35]. Animals were kept in a tie-stall barn under 18:30 h of light per day (05:00 to 23:30 h) and milked twice daily (07:30 and 19:30 h). The experimental period began 3 weeks before the expected calving date and lasted until 9 weeks of lactation. The cows were fed ad libitum a close-up diet beginning 3 weeks before the expected date of calving until parturition and, then a lactation diet both formulated to meet or exceed the National Research Council (NRC) recommendations [36]. Long hay (0.5 kg) was given at 07:30 h and total mixed ration was served once daily at 08:30 h. Cows had free access to water.

Cows were assigned to 6 blocks of 4 animals each according to their 305-d milk production during the previous lactation. Within each block, cows were randomly assigned to one of the following treatments: weekly intramuscular injections of 5 mL of either saline 0.9 % NaCl (Control group), 320 mg of pteroylmonoglutamic acid (MP Biomedicals, Solon, OH, USA; (Vitamin B9 group), 10 mg of cyanocobalamin (5 000 μg/mL, Vetoquinol, Lavaltrie, Quebec, Canada; (Vitamin B12 group) or 320 mg of pteroylmonoglutamic acid and 10 mg of cyanocobalamin (B9 + B12 group). Thus, there were 6 animals per treatment group.

### Biological material collection and tissue handling

Mammary gland and hepatic tissues were obtained from the lactating dairy cows, 64 ± 3 days after calving. Biopsies were performed under local anesthesia. The process of hepatic biopsies uses ultrasound guidance to minimize the hemorrhagic risks [4]. Both procedures were approved by the Institutional Committee on Animal Care of the Sherbrooke Research and Development Centre, Agriculture and Agri-Food Canada, Sherbrooke, QC, Canada according to the guidelines of the Canadian Coucil on Animal Care [37]. Tissues were immediately frozen into liquid nitrogen and stored at −80 °C until use.

### Total RNA isolation and purification

Total RNA was extracted from hepatic and mammary tissues by using a QIAzol Lysis Reagent (QIAGEN Inc., Toronto, ON, Canada) following the original manufacturer’s protocol, with slight modifications. Briefly, frozen samples (100 mg of tissue) were homogenized in 2 mL of QIAzol Lysis Reagent on ice using a Tissue-Tearor. A volume of 600 μL QIAzol Lysis Reagent was added to 400 μL of homogenate; the mixture was vigorously vortexed and kept at room temperature for 5 min to promote dissociation of nucleoprotein complexes. A volume of 200 μL of chloroform was added; the mixture was shaken and left at room temperature for 3 min followed by a centrifugation at 12 000 × g for 15 min at 4 °C to remove lipids. After centrifugation, the aqueous fraction (upper layer) was taken and RNA was precipitated by adding an equal volume of 70 % ethanol. RNA was purified according to manufacturer’s procedure using RNeasy Mini Kit (QIAGEN Inc., Toronto, ON, Canada), including on-column DNase digestion. The purity, concentration, and integrity of total RNA intended for qPCR were assessed. Purity of the RNA was evaluated by absorbance (A) readings (ratio of A260/A230 and A260/A280) using a NanoDrop ND-1000 spectrophotometer (Thermo Fisher Scientific, Waltham, MA, US). NanoDrop ND-1000 spectrophotometer was also used to measure the concentration. All RNA samples passed the quality control. The RNA Integrity Number calculated by the Bioanalyzer software at McGill University and Génome Québec Innovation Center (Montreal, Quebec, Canada) ranged from 7.3 to 8.7.

### Microarray

McGill University and Génome Québec Innovation Center (Montreal, Quebec, Canada) performed the microarray analysis. Cyanine 3-labeled CTP complementary RNA (cRNA) was produced with 50 ng of total RNA using the Low Input Quick Amp Labeling Kit, according to manufacturer’s instructions (Agilent Technologies, Inc). The quality of cRNA was evaluated by capillary electrophoresis on 2100 Electrophoresis Bioanalyzer instrument (Agilent technologies, Santa Clara, CA, USA). A total of 15 525 genes were analyzed via expression levels of 42 789 probes using the Agilent Bovine Genome Oligo Microarrays 4 × 44 K (G2519F-023647) (Agilent technologies, Santa Clara, CA, USA). Labeling, hybridization, and raw data extraction were performed by McGill University and Génome Québec Innovation Center (Montreal, Quebec, Canada) according to the manufacturer’s instructions, as previously described [38]. Hybridizations were performed by batch with samples randomly distributed. The hybridizations of microarrays were compared through a correlation matrix that enables the quick identification of poor and divergent replication (data not shown). Once the slides are scanned, the respective “.tif” image was examined using the Agilent Feature Extraction (FE) software. Scan image information is displayed in the Scan Image Properties for images that were generated using the Agilent Scanner. Then data are extracted with the FE software. A quality control (QC) report is generated for each sample. The FE version 10.7.3.1 with GE1_107_Sep09 protocol and grid associated with the selected type of chip 023647_D_F_20110614 were used. All microarray datasets passed all the quality criteria and were then downloaded into the FlexArray microarray analysis software (http://gqinnovationcenter.com/documents/technicalNotes/technicalNotes_GQ06.pdf). Array data have been submitted to the public databases and assigned Gene Expression Omnibus (GEO) accession number is GSE77421.

FlexArray, a Bioconductor R based software, was developed by Génome Québec to provide researchers with a user-friendly interface for the analysis of microarray experiments. Raw microarray expression intensities were corrected for background using normexp, according to Ritchie et al. [39]. Between-array normalization was performed so that the background corrected intensities have similar distributions across the arrays. Comparison of the vitamin treatments with the control tissue was performed by Cyber-T, a version of the *t*-test that uses a Bayesian estimate of the within treatment variance [40, 41]. Up- or downregulated genes lists were analyzed on Protein ANalysis THrough Evolutionary Relationships (Panther) classification system [42].

### Quantitative real time-PCR

Quantifications by real-time PCR (qPCR) following reverse transcription were performed as previously described [43] with minor modifications. The reverse transcription PCR reactions were performed with the SuperScript II reverse transcriptase (Life Technologies Inc., Burlington ON, Canada) according to the manufacturer’s protocol and using 500 ng of total RNA extracted from each of the 24 animals. An equivalent quantity of cDNA is synthesized in a final reaction volume of 20 μL, giving a concentration of 25 ng/μl of cDNA. A pool of cDNA intended to estimate the efficiencies primers, was made using the cDNA of each animal. Primers were designed for each gene using the Primer Express 3 software package (Applied Biosystems, Life Technologies Corporation, Burlington, ON, Canada) using the reference sequence from the RefSeq database of the National Center for Biotechnology Information depository. Primers for a total of 34 genes for both tissues were designed. Optimizations of primers were performed for each gene by testing different concentrations of both forward and reverse primers, each ranging from 50 to 900 nM. Estimations of primer efficiencies were analyzed using the standard curves made from a serial of seven dilutions (1/7.5, 1/15, 1/30, 1/60, 1/120, 1/240, 1/480) of the pool of the cDNA samples (25 ng/μl of cDNA). As 3 μl of each dilution were used in a final reaction volume of 10 μl for qPCR, the concentrations used per dilution were: 1, 0.5, 0.25, 0.125, 0.0625, 0.03125 and 0.015625 (ng/μl). Additional file 1 provides experimental information and PCR amplification efficiency for all genes. The qPCR reactions (10 μL, final volume) were performed on 96 well plates using Fast SYBR Green PCR Master Mix (Applied Biosystems) in a 7500 Fast Real Time-PCR System (Life Technologies, Burlington, ON, Canada) as the manufacturer’s instructions. The PCR thermal cycling conditions comprised an initial 20 s denaturation step at 95 °C followed by 40 cycles at 95 °C for 3 s followed by an annealing/elongation period at 60 °C for 30 s. A dissociation step was included for all amplifications to confirm the presence of single discrete PCR products of the expected size. Twenty-five genes (14 from hepatic tissue data and 11 from mammary gland tissue data) were subject to qPCR validation because they were found differentially expressed by microarray as expressed by log2 of their fold change. In addition, in the mammary gland, four genes (*FOLR1*, *ELF5*, *B4GALT1* and *LALBA*) were chosen because of their implication in the metabolic pathway involving vitamins B9 and B12. The expression of 5 putative reference genes, namely actin beta (*ACTB*), ubiquitously-expressed transcript (*UXT*), peptidylprolyl isomerase A (*PPIA*), glyceraldehyde-3-phosphate dehydrogenase (*GAPDH*), tyrosine 3-monooxygenase/tryptophan 5-monooxygenase activation protein, zeta polypeptide (*YWHAZ*) was determined for all samples as recommended [44–46]. All the 24 animals were used to perform the qPCR analysis of 34 genes for both tissues. Once the more stable genes were selected, the normalization factor was calculated using a geometrical average as recommended [45]. The combination of *ACTB* and *PPIA* was appropriate to normalize the data from hepatic tissue. The appropriated combination was *UXT* and *PPIA* to normalize the data from mammary gland.

### Data analysis and statistics

Using FlexArray microarray analysis, a significant result at a *P* value < 0.05 after false discovery rate correction with a minimum of ± two-fold change in gene expression for the respective treatment group compared to control tissues was considered biologically interesting. Gene category over-representation analysis consists in grouping genes into categories by some common biological property and then tested to find categories that are over represented amongst the differentially expressed genes. Gene ontology describes and categorizes gene products in three non-overlapping domains of molecular biology [47]. Panther (http://pantherdb.org) is a visualisation browser of GO [48], using version 10 which included other organisms. Panther ranks proteins (and their genes) according to Family (and subfamily), Molecular function, Biological process and Pathway. The process of classification is extensively explained by Mi and colleagues [42]. Only significant differentially expressed genes were analysed by qPCR. Data from qPCR were analyzed using SAS Institute procedures (2008). Means were assumed to be different at *P* ≤ 0.05 and tended to differ at 0.05 < *P* ≥ 0.1. Normfinder indicated the interested combination of reference genes to normalize qPCR data.

## Abbreviations

ECM, extracellular matrix components; GO, gene ontology; HPCs, hepatic progenitor cells; Panther, protein analysis through evolutionary relationships; qPCR, quantitative polymerase chain reaction.

## Additional file

Additional file 1: Oligonucleotide primer sequences for quantitative PCR. (DOCX 32 kb)

## References

[1] Lardinois CC, Mills RC, Elyehjem CA, Hart EB (1944). Rumen synthesis of the vitamin B complex as influenced by ration composition. J Dairy Sci.

[2] Girard CL, Matte JJ (2005). Effects of intramuscular injections of vitamin B12 on lactation performance of dairy cows fed dietary supplements of folic acid and rumen-protected methionine. J Dairy Sci.

[3] Preynat A, Lapierre H, Thivierge MC, Palin MF, Matte JJ, Desrochers A (2009). Effects of supplements of folic acid, vitamin B12, and rumen-protected methionine on whole body metabolism of methionine and glucose in lactating dairy cows. J Dairy Sci.

[4] Graulet B, Matte JJ, Desrochers A, Doepel L, Palin MF, Girard CL (2007). Effects of dietary supplements of folic acid and vitamin B12 on metabolism of dairy cows in early lactation. J Dairy Sci.

[5] Scott JM (1999). Folate and vitamin B12. Proc Nutr Soc.

[6] Girard CL, Matte JJ, Tremblay GF (1995). Gestation and lactation of dairy cows: a role for folic acid?. J Dairy Sci.

[7] Girard CL, Matte JJ (1998). Dietary supplements of folic acid during lactation: effects on the performance of dairy cows. J Dairy Sci.

[8] Menzies KK, Lefevre C, Macmillan KL, Nicholas KR (2009). Insulin regulates milk protein synthesis at multiple levels in the bovine mammary gland. Funct Integr Genomics.

[9] Duplessis M. Impact d’un supplément combine d’acide folique et de vitamine B12 en période prépartum et en début de lactation chez la vache laitière. Université Laval 2014. PhD Thesis. Univ. Laval, Quebec, QC, Canada, p. 301.

[10] McEwen M, Reynolds K (2006). Noninvasive detection of bilirubin using pulsatile absorption. Australas Phys Eng Sci Med.

[11] Larsen M, Kristensen NB (2013). Precursors for liver gluconeogenesis in periparturient dairy cows. Animal.

[12] Amaral DM, Veenhuizen JJ, Drackley JK, Cooley MH, McGilliard AD, Young JW (1990). Metabolism of propionate, glucose, and carbon dioxide as affected by exogenous glucose in dairy cows at energy equilibrium. J Dairy Sci.

[13] Danfaer A, Tetens V, Agergaard N (1995). Review and an experimental study on the physiological and quantitative aspects of gluconeogenesis in lactating ruminants. Comp Biochem Physiol B Biochem Mol Biol.

[14] Nafikov RA, Beitz DC (2007). Carbohydrate and lipid metabolism in farm animals. J Nutr.

[15] Grummer RR (1995). Impact of changes in organic nutrient metabolism on feeding the transition dairy cow. J Anim Sci.

[16] McCarthy SD, Waters SM, Kenny DA, Diskin MG, Fitzpatrick R, Patton J (2010). Negative energy balance and hepatic gene expression patterns in high-yielding dairy cows during the early postpartum period: a global approach. Physiol Genomics.

[17] Connor EE, Siferd S, Elsasser TH, Evock-Clover CM, Van Tassell CP, Sonstegard TS (2008). Effects of increased milking frequency on gene expression in the bovine mammary gland. BMC Genomics.

[18] Mi H, Muruganujan A, Casagrande JT, Thomas PD (2013). Large-scale gene function analysis with the PANTHER classification system. Nat Protoc.

[19] Jelnes P, Santoni-Rugiu E, Rasmussen M, Friis SL, Nielsen JH, Tygstrup N (2007). Remarkable heterogeneity displayed by oval cells in rat and mouse models of stem cell-mediated liver regeneration. Hepatology.

[20] Falix FA, Aronson DC, Lamers WH, Gaemers IC (2012). Possible roles of DLK1 in the Notch pathway during development and disease. Biochim Biophys Acta.

[21] Zhu NL, Asahina K, Wang J, Ueno A, Lazaro R, Miyaoka Y (2012). Hepatic stellate cell-derived delta-like homolog 1 (DLK1) protein in liver regeneration. J Biol Chem.

[22] Tsukamoto H (2015). Metabolic reprogramming and cell fate regulation in alcoholic liver disease. Pancreatology.

[23] Li H, Cui ML, Chen TY, Xie HY, Cui Y, Tu H (2015). Serum DLK1 is a potential prognostic biomarker in patients with hepatocellular carcinoma. Tumour Biol.

[24] Pan RL, Xiang LX, Wang P, Liu XY, Nie L, Huang W (2015). Low-molecular-weight fibroblast growth factor 2 attenuates hepatic fibrosis by epigenetic down-regulation of Delta-like1. Hepatology.

[25] Lee YH, Yun MR, Kim HM, Jeon BH, Park BC, Lee BW (2016). Exogenous administration of DLK1 ameliorates hepatic steatosis and regulates gluconeogenesis via activation of AMPK. Int J Obes (Lond).

[26] Busnadiego O, Gonzalez-Santamaria J, Lagares D, Guinea-Viniegra J, Pichol-Thievend C, Muller L (2013). LOXL4 is induced by transforming growth factor beta1 through Smad and JunB/Fra2 and contributes to vascular matrix remodeling. Mol Cell Biol.

[27] Hayashi K, Fong KS, Mercier F, Boyd CD, Csiszar K, Hayashi M (2004). Comparative immunocytochemical localization of lysyl oxidase (LOX) and the lysyl oxidase-like (LOXL) proteins: changes in the expression of LOXL during development and growth of mouse tissues. J Mol Histol.

[28] Yang X, Zhang X, Heckmann BL, Lu X, Liu J (2010). Relative contribution of adipose triglyceride lipase and hormone-sensitive lipase to tumor necrosis factor-alpha (TNF-alpha)-induced lipolysis in adipocytes. J Biol Chem.

[29] Yang X, Lu X, Lombes M, Rha GB, Chi YI, Guerin TM (2011). The G(0)/G(1) switch gene 2 regulates adipose lipolysis through association with adipose triglyceride lipase. Cell Metab.

[30] Gessner DK, Schlegel G, Keller J, Schwarz FJ, Ringseis R, Eder K (2013). Expression of target genes of nuclear factor E2-related factor 2 in the liver of dairy cows in the transition period and at different stages of lactation. J Dairy Sci.

[31] Hsiao CP, Araneta M, Wang XM, Saligan LN (2013). The association of IFI27 expression and fatigue intensification during localized radiation therapy: implication of a para-inflammatory bystander response. Int J Mol Sci.

[32] Qian P, He XC, Paulson A, Li Z, Tao F, Perry JM (2016). The Dlk1-Gtl2 Locus Preserves LT-HSC Function by Inhibiting the PI3K-mTOR Pathway to Restrict Mitochondrial Metabolism. Cell Stem Cell.

[33] Bobe G, Young JW, Beitz DC (2004). Invited review: pathology, etiology, prevention, and treatment of fatty liver in dairy cows. J Dairy Sci.

[34] Katoh N (2002). Relevance of apolipoproteins in the development of fatty liver and fatty liver-related peripartum diseases in dairy cows. J Vet Med Sci.

[35] NFACC (2009). Code of practice for the care and handling of dairy cattle.

[36] NRC. Nutrient Requirements of Dairy Cattle (2001). Seventh Revised Edition, 2001.

[37] CCAC (2003). CCAC guidelines on: laboratory animal facilities - characteristics, design, and development.

[38] Rudkowska I, Paradis AM, Thifault E, Julien P, Tchernof A, Couture P (2013). Transcriptomic and metabolomic signatures of an n-3 polyunsaturated fatty acids supplementation in a normolipidemic/normocholesterolemic Caucasian population. J Nutr Biochem.

[39] Ritchie ME, Silver J, Oshlack A, Holmes M, Diyagama D, Holloway A (2007). A comparison of background correction methods for two-colour microarrays. Bioinformatics.

[40] Baldi P, Long AD (2001). A Bayesian framework for the analysis of microarray expression data: regularized t -test and statistical inferences of gene changes. Bioinformatics.

[41] Kayala MA, Baldi P (2012). Cyber-T web server: differential analysis of high-throughput data. Nucleic Acids Res.

[42] Mi H, Poudel S, Muruganujan A, Casagrande JT, Thomas PD (2016). PANTHER version 10: expanded protein families and functions, and analysis tools. Nucleic Acids Res.

[43] Levesque-Sergerie JP, Duquette M, Thibault C, Delbecchi L, Bissonnette N (2007). Detection limits of several commercial reverse transcriptase enzymes: impact on the low- and high-abundance transcript levels assessed by quantitative RT-PCR. BMC Mol Biol.

[44] Lisowski P, Pierzchala M, Goscik J, Pareek CS, Zwierzchowski L (2008). Evaluation of reference genes for studies of gene expression in the bovine liver, kidney, pituitary, and thyroid. J Appl Genet.

[45] Bionaz M, Loor JJ (2007). Identification of reference genes for quantitative real-time PCR in the bovine mammary gland during the lactation cycle. Physiol Genomics.

[46] Kadegowda AK, Bionaz M, Thering B, Piperova LS, Erdman RA, Loor JJ (2009). Identification of internal control genes for quantitative polymerase chain reaction in mammary tissue of lactating cows receiving lipid supplements. J Dairy Sci.

[47] Harris MA, Clark J, Ireland A, Lomax J, Ashburner M, Foulger R (2004). The Gene Ontology (GO) database and informatics resource. Nucleic Acids Res.

[48] Thomas PD, Campbell MJ, Kejariwal A, Mi H, Karlak B, Daverman R (2003). PANTHER: a library of protein families and subfamilies indexed by function. Genome Res.

