# Supplementary material for: Supplements of vitamins B9 and B12 affect hepatic and mammary gland gene expression profiles in lactating dairy cows
Source: BMC Genomics. 2016 Aug 15;17:640. doi: 10.1186/s12864-016-2872-2 (PMC4986251; doi:10.1186/s12864-016-2872-2)
Supplement: Additional file 1: — Oligonucleotide primer sequences for quantitative PCR. (DOCX 32 kb) [file 12864_2016_2872_MOESM1_ESM.docx]

Oligonucleotide primer sequences for real time-PCR

**Additional file 1**

| Gene name | Accession number | Primer ID | Primer sequence (5’-3’) | Efficiency (%) | Primer (nM) | Size (bp) |
| --- | --- | --- | --- | --- | --- | --- |
| *ACTB* | NM_173979 | F1051 | TGGCACCCAGCACAATGA | 92.4 | 300/300 | 123 |
|  |  | R1173 | CCTGCTTGCTGATCCACATCT |  |  |  |
| *B4GALT1* | NM_177512 | F1125 | GGGAGGTGAAGATGATGACATTTAT | 96.3 | 300/300 | 88 |
|  |  | R1212 | CCGACACTTCCCGATCACA |  |  |  |
| *CIDEA* | NM_001083449 | F536 | CAGGGTTCAAGGCCATGCT | 87.4 | 300/300 | 91 |
|  |  | R627 | GTAGGTGCCCATGTGGATAAGAC |  |  |  |
| *DLK1* | NM_174037 | F970 | AGAACCTGCTGCTGCACTACAA | 76.9 | 300/300 | 121 |
|  |  | R1091 | GGGCGCTGCTCAGATCTC |  |  |  |
| *ELF5* | NM_001024569 | F557 | AACAAGTGGCATCAAAAGTCAAGA | 85.6 | 300/300 | 81 |
|  |  | R637 | CGCACAAATTCCCATAGATGAGA |  |  |  |
| *FOLR1* | NM_001206532 | F529 | GAAGACTGCCGCACCTCCTA | 86.9 | 900/300 | 89 |
|  |  | R617 | GCTTTCACTGGGCACTGGTT |  |  |  |
| *G0S2* | NM_001192147 | F606 | CCACGTCCAAAGCTAGGTACCT | 89.5 | 50/300 | 68 |
|  |  | R673 | TCCTTTCACCCTCGTGCAA |  |  |  |
| *GPNMB* | NM_001038065 | F2504 | AGGGTCAGTACTCGTTCATTCATTTT | 99.8 | 900/900 | 96 |
|  |  | R2600 | TTCAGGTATGTCCACAAGATTGTCA |  |  |  |
| *GPR110* | NM_001192327 | F2373 | TACGGGTGCCCTCTCATTATATC | 84.4 | 300/300 | 99 |
|  |  | R2471 | ATCGGACCAGTTAAGCCAACAT |  |  |  |
| *GSTA5* | NM_001099016 | F454 | AACAAACCGTTATCTCCCTGCAT | 101.9 | 300/300 | 90 |
|  |  | R544 | GATGTCAGCCTTGCTCAGCTT |  |  |  |
| *H1FNT* | XM_002687347 | F346 | GCCATCGCCTCACACAAAA | 115.8 | 300/300 | 71 |
|  |  | R417 | CACCTCGTAGCCCGCATT |  |  |  |
| *HERC6* | NM_001192644 | F1169 | TTGCTGGAACATATGCCAACTT | 80.9 | 300/300 | 127 |
|  |  | R1295 | GCCACTGCCATCCATTTTTC |  |  |  |
| *HP* | NM_001040470 | F637 | CACAGTAGTGACAAAAAAGCAAAGG | 72.6 | 50/300 | 107 |
|  |  | R744 | GGAGTGGTCAGGGTGGAGAAC |  |  |  |
| *IFI27* | NM_001038050 | F600 | GCGGCCAAGATGATGTCAATA | 79.8 | 300/300 | 119 |
|  |  | R718 | AAGAGTTTGGATGACAGGCAGAGT |  |  |  |
| *ISG15* | NM_174366 | F408 | GACCAGTTCTGGCTGTCTTTTGA | 97.5 | 300/300 | 112 |
|  |  | R519 | CACCCCGAAGACGTAGATTCAT |  |  |  |
| *LALBA* | NM_174378 | F284 | CTCACTCAAGCAACATCTGTAACATC | 108.9 | 300/300 | 129 |
|  |  | R412 | GTGCTTTATGGGCCAACCA |  |  |  |
| LOC785756 | NM_001206806 | F230 | GATGCCAAGCTGAAAGTTGTAGAAA | 99.0 | 300/300 | 88 |
|  |  | R318 | CTGCTCAGAACCGTGGACACT |  |  |  |
| *LOXL4* | NM_174384 | F1963 | CAGGCACTACCACAGCATTGAG | 97.6 | 300/300 | 137 |
|  |  | R2099 | CATAGCGCCTTTGCATTCCT |  |  |  |
| *MEP1B* | NM_001144098 | F1908 | GTACATGGGAGAAAGGTGTGAAAA | 84.8 | 300/900 | 116 |
|  |  | R2023 | AGACGCTGACGAGAGTGATGATC |  |  |  |
| *MT1A* | NM_001040492 | F187 | TGTGGGCTGTGCCAAGTGT | 75.3 | 300/300 | 105 |
|  |  | R292 | TCTATTGACAACTAGGGCAGGTTCT |  |  |  |
| *POSTN* | NM_001040479 | F1209 | CCTGATTCTGCCAAACAAGTTATTG | 96.0 | 900/300 | 92 |
|  |  | R1301 | CAGAGCAGATGCCAAGCCTAA |  |  |  |
| *PPIA* | NM_178320 | F317 | ATGCTGGCCCCAACACAA | 89.0 | 300/300 | 101 |
|  |  | R417 | CCCTCTTTCACCTTGCCAAA |  |  |  |
| *PPP1R3B* | NM_001103247 | F830 | TGCAGTCTACCCAGGGAACAG | 78.8 | 300/900 | 118 |
|  |  | R947 | AAATAACTAGGCCACTCCGGAAAC |  |  |  |
| *RAB15* | NM_001046538 | F2930 | CTGGGATTTTAGGGTGCAGCTA | 86.5 | 300/300 | 113 |
|  |  | R3042 | CACGCCTGCTCAACAGTCTAAG |  |  |  |
| *SFRP1* | NM_174460 | F1244 | GGAGGGCGGCGTAACC | 67.2 | 900/300 | 104 |
|  |  | R1348 | TGGGCGTGCGGCTTTA |  |  |  |
| *SLC22A9* | NM_001046006 | F2090 | AAAAATGAAGTTAATGGAGGGAAGGT | 95.8 | 300/300 | 101 |
|  |  | R2191 | CCTATTCTGGATGTCTTTTGAGTTGTAA |  |  |  |
| *SPP1* | NM_174187 | F507 | AAATGATGGCCGAGGTGATAGT | 97.6 | 300/300 | 146 |
|  |  | R652 | TCTTAGGTGCGTCATGCATCTC |  |  |  |
| *UXT* | NM_001037471 | F339 | TGGCAGAAGCTCTCAAGTTCATT | 95.7 | 300/300 | 105 |
|  |  | R443 | CATGTGGATATGGGCCTTGAT |  |  |  |

All of the genes analyzed by RT-PCR in liver or mammary gland are listed. The combination of data from the housekeeping genes *ACTB* and *PPIA* was used to normalize the data of targeted genes in hepatic tissue while *PPIA* and *UXT* normalized the data of targeted genes in mammary tissue.
